# Investigating behavioural addictions in adults with and without attention deficit hyperactivity disorder

**DOI:** 10.1371/journal.pone.0317525

**Published:** 2025-02-05

**Authors:** James L. Findon, Annika Muck, Beáta Tóthpál- Davison, Eleanor J. Dommett

**Affiliations:** Department of Psychology, Institute of Psychiatry, Psychology and Neuroscience, London, United Kingdom; City University of New York, UNITED STATES OF AMERICA

## Abstract

Attention deficit hyperactivity disorder (ADHD) confers greater risk of alcohol and substance use disorders, which may be linked to altered compulsivity and impulsivity within the condition. However, no study has investigated the link between these constructs and behavioural addictions in ADHD. The aim of this study was to investigate whether individuals with ADHD show increased impulsivity, compulsivity, and associated distress, as well as addictive behaviour for gambling, exercise, and internet use, and to assess the relationship between these factors. Adults with and without ADHD were recruited from across the UK and completed an online survey measuring impulsivity, compulsivity, gambling, internet use and exercise addiction. Three hundred and forty-six adults took part (Healthy control = 137, ADHD-medicated = 110, ADHD-unmedicated = 99). Those declaring a diagnosis of ADHD reported greater internet use problems and greater withdrawal from exercise. Medicated individuals also reported higher exercise continuance and were more likely be symptomatic for exercise dependency. Individuals identifying with ADHD had greater levels of impulsivity and compulsivity, with impulsivity correlated with all behavioural addictions, whilst compulsivity correlated only with exercise and internet use. Regression analyses indicated that the distress caused by impulsivity and compulsivity was associated with internet use. Although further research is needed, this study indicates that the greater risk of behavioural addictions found in those with ADHD may relate to impulsivity and compulsivity, and that this should be considered when managing ADHD.

## Introduction

Attention deficit hyperactivity disorder (ADHD) is characterised by symptoms of inattention, and/or hyperactivity and impulsivity, resulting in significant functional impairment [1]. Although initially conceived as a childhood condition, it is now recognised to impact adults with a global prevalence of 2.58% [2]. Adults with ADHD report lower quality of life [3] and exhibit several negative outcomes including lower levels of post-compulsory education, less occupational stability, lower income and more state financial support and greater emotional difficulties than individuals without ADHD [4].

ADHD is associated with a range of comorbidities, with alcohol and substance use disorders being amongst the most common [5–7]. Exactly why ADHD confers a greater risk of these disorders is not clear. It has been suggested that use of psychostimulant medication, the first-line treatment for the condition [8], could contribute to this risk but this has yet to be supported by meta-analyses [9] and there is evidence to suggest that the risk of alcohol and substance use disorder is actually lower in those receiving medication compared to those with untreated ADHD [10, 11]. Alternative explanations centre around individuals with ADHD exhibiting more risky decision making [12] and, as would be expected from the symptom profile, higher impulsivity [6]. Impulsivity is defined as actions which are “poorly conceived, prematurely expressed, unduly risky or inappropriate” [13] and impulsive behaviours are ultimately driven by the promise of a reward. Within the context of addiction, impulsivity is considered a risk factor for initiating use of addictive substances as well as being implicated in progression to dependence, persistence and relapse [14]. Alongside impulsivity, a role for compulsivity has been identified in addiction, particularly in the later stages where behaviour is driven by a need to avoid negative experiences [15]. Compulsivity can be defined as repetitive actions, often driven by a feeling that one has to act [16] which are “inappropriate to the situation, [and] have no obvious relationship to the overall goal” [17]. Although ADHD is not typically considered a compulsive disorder, it is thought to sit within a spectrum of impulsive-compulsive disorders [18], and it is strongly associated with both obsessive-compulsive disorder [19] and hoarding disorder [20]. Therefore, it is possible that both the heightened impulsivity and altered compulsivity explain some of the elevated risk of alcohol and substance use disorders in those with ADHD. This is supported by research indicating that those with ADHD deemed high risk for such disorders have an imbalance between inhibitory control and motivation-reward processing neural networks [6]. These circuits are implicated in addiction and it is accepted that a tendency to act impulsively and compulsively, which is also linked to these neural circuits [21], are core features of addiction [22].

In addition to the increased risk of alcohol and substance use disorders in ADHD, there is evidence for increased risk of behavioural addictions. Studies have identified increased risk for accepted and proposed behavioural addictions in ADHD including gambling [23–25], internet addiction [26, 27], gaming addiction [28], compulsive buying [29] and exercise addiction [30, 31], the latter of which is particularly controversial, given that exercise has been proposed as an intervention for ADHD management [8]. The risk of exercise addiction may also differ depending on whether the individual is medicated or not, as previous research has found a greater risk among individuals with ADHD on medication [30].

Evidence suggests that impulsivity and compulsivity may be important in these behavioural addictions. A meta-review revealed that gambling disorder, the only formally recognised behavioural addiction, was consistently associated with impulsivity and compulsivity deficits across a range of tasks. However, none of the studies included in the review incorporated participants with ADHD. More recent work which examined a range of potential behavioural addictions (gaming, internet use, exercise, shopping and work) alongside gambling reported that compulsivity dominated over impulsivity in more severe behavioural addictions [32] but this study did not include any individuals with ADHD.

Some studies have investigated the relationship between behavioural addictions and impulsivity in ADHD, showing that greater impulsivity corresponds to higher scores on addiction measures or risk of addiction, for gaming [33], internet use [34] and gambling [23, 24, 35]. However, to date no studies have investigated the relationship between impulsivity, compulsivity, and behavioural addictions in ADHD. Therefore, given the current gaps in the literature, the aim of the present study was to examine the traits of impulsivity and compulsivity within an ADHD group, and a healthy control group for comparison purposes, and to assess the relationship between these measures and different behavioural addictions. Specifically, we hypothesized that i) those with ADHD will show increased levels of impulsivity, compulsivity, as well as the distress caused by these, and addictive behaviours for gambling, exercise, and internet use (H1), and ii) addiction scores will be associated with levels of impulsivity and compulsivity, and their associated distress (H2).

## Methods

### Participants

To be eligible to participate, individuals had to be currently living in the United Kingdom and aged 18 years or older. Healthy controls were required to be free of psychiatric and neurological conditions. Participants in the ADHD group were required to confirm that they had an existing diagnosis of ADHD made by a clinician. Participation was voluntary and participants were recruited using convenience sampling through online advertising (Facebook, Instagram, university newsletters), where they were invited to take part in an anonymous online survey hosted on the platform Qualtrics. Adverts provided a link to the information sheet, including contact details for the researchers, and an online consent form, which had to be completed prior to the survey questions being accessed. Given the focus on potentially addictive behaviours, the survey provided details of relevant support services throughout to ensure that even those choosing not to complete the survey were signposted to support. All participants were then given a downloadable resource signposting, as a minimum, NHS support for the specific conditions identified. Participation was incentivised with the option of entering a prize draw for shopping vouchers (prizes of £25, £50, and £100). To enter the prize draw, participants provided an email address that could not be linked to their research data via a separate survey after completing the research study. The authors assert that all procedures contributing to this work comply with the ethical standards of the relevant national and institutional committees (King’s College London HR/DP-21/22-28512) on human experimentation and with the Helsinki Declaration of 1975, as revised in 2008.

### Survey: Procedure and measures

Data collection was conducted between 26^th^ February 2022 and 3^rd^ May 2023. After consent was provided, at the start of the survey, inclusion criteria were checked by asking participants to confirm that they currently lived in the United Kingdom, were over 18 years old, and either held a current ADHD diagnosis or were free of any psychiatric or neurological conditions, as required for the control group. Following this, participants were asked to provide demographic information from pre-specified response options (gender, age, ethnicity and level of education attained). They were also asked to indicate the presence of any learning differences selecting from none, dyslexia, dyscalculia, specific learning difference (SLD), or the option to enter a free text response. Those declaring that they had an ADHD diagnosis from a healthcare professional were further asked to select their medication status and whether they received any other treatments for ADHD (with a free text option to indicate type of medication and treatment). Those receiving medication were asked to indicate adherence using a previously modified adherence measure [36]. These details were collected to allow for characterisation of the sample, but also to separate medicated and unmedicated individuals, given that previous work has indicated different levels of risk of addiction [10, 11, 30]. Participants were also asked about the presence of any psychiatric or neurological conditions, as well as treatments they are receiving for them via free text options. Following this, several standardised measures were included to assess ADHD, impulsivity, and compulsivity.

#### Adult ADHD Self-Report Scale (ASRS)

The 18-item ASRS is based on the Diagnostic Statistical Manual of Mental Disorders-IV (DSM-IV) Criterion A symptoms of adult ADHD [37]. It uses a 5-point Likert scale (0 = never, 4 = very often). The scale be divided into two 9-item subscales measuring inattention and hyperactivity/impulsivity. Additionally, part A (first six items—screener) is considered to have good predictive abilities for a clinical diagnosis of ADHD with a cut-off score of 14 [37–39]. The scale has good specificity but lower sensitivity [39] and is proposed to work well for research purposes [40], including general population samples [40–42]. In our study, all elements of the ASRS had excellent internal consistency (18-items α = 0.97, Screener α = 0.91, Inattention (IA) α = 0.95 and hyperactivity-impulsivity (HI) α = 0.93). A full breakdown of Cronbach’s alpha values for the different subgroups (Healthy Control, ADHD-Medicated and ADHD-unmedicated) within our sample is provided in S1 Table.

#### Impulsive-Compulsive Behaviours Checklist (ICBC)

The 33-item Impulsive-Compulsive Behaviours Checklist (ICBC) assesses problematic impulsive and compulsive behaviours and the distress they cause [43]. It lists 33 behaviours, e.g., collecting, self-harm, and alcohol consumption, and scores them on a 4-point Likert scale (1 = never, 4 = always). The ICBC has two subscales–impulsive compulsions (IC, compulsive behaviours), and compulsive-impulsions (CI, impulsive behaviours) with good internal consistency [43]. Additionally, for each item, participants indicate (yes/no) whether each behaviour causes distress which is summed to give a distress score. In our study, the ICBC had very good internal consistency (Total α = 0.93, IC α = 0.91, CI α = 0.86). Subgroup internal consistencies are available in S1 Table.

#### Barratt Impulsiveness Scale-15 (BIS-15)

The BIS-15 was used to measure impulsivity with ratings on a 4-point Likert scale (1 = rarely/never, 4 = almost/always). The BIS-15 has good internal consistency and test-retest reliability [44]. In this study, internal reliability was very good (α = 0.86) (see S1 Table for subgroup reliability).

Respondents were then asked to indicate which of three behaviours (exercise, internet use, gambling) they had engaged in at least once within the last six months. Depending on which behaviours the participants engaged in, they then received the corresponding questionnaire(s) to complete.

#### Exercise Dependence Scale-Revised (EDS-R)

The 21-item Exercise Dependence Scale-Revised (EDS-R) assesses exercise addiction. Items are scored on a 6-point Likert scale (1 = never, 6 = always). It contains seven subscales (tolerance, withdrawal, continuance, lack of control, reduction in other activities, time, and intention) and can also be used to calculate an ‘exercise dependence risk’ score, which is made up of three categories–‘at risk of exercise dependence’, ‘symptomatic non-dependent’ (shows symptoms of exercise addiction, but is not dependent), and ‘asymptomatic non-dependent’ (does not show symptoms). Good internal consistency and 1-week test-retest reliability have been demonstrated [45]. In our study, the EDS-R had an internal consistency of α = 0.95 with all subscales reliable (α ≥ 0.75; see S1 Table for subgroup reliability).

#### Brief Problem Gambling Screen-5 (BPGS-5)

The BPGS-5 is a screening tool to identify problematic gambling with good classification accuracy, with a sensitivity of 95% for men and 97% for women. Items are coded using binary (0 = no; 1 = yes [46]). In our study, the BPGS-5 had an internal consistency of α = 0.74 (see S1 Table for subgroup reliability).

#### Short Compulsive Internet Use Scale (Short CIUS)

The 5-item CIUS uses a 5-point Likert scale (0 = never, 4 = always) and has demonstrated good internal consistency, sensitivity (95%), and specificity (87%; [47]). In our study, it had an internal consistency of α = 0.89 (see S1 Table for subgroup reliability).

### Data processing and analyses

The data analyses were performed using SPSS (Version 28.0; IBM Corp, 2020). In total, 697 participants consented to the study and accessed the survey. Before data analysis, several checks and exclusions were made. Four participants were removed because they had not provided an age, meaning we could not confirm eligibility. Given our reliance on self-report of an existing ADHD diagnosis, we used the ASRS-A scores to confirm inclusion within specific groups. Healthy controls who scored ≥14 on the ASRS-A were excluded (241 excluded, 58.9% of all healthy controls). A further 31 individuals in the healthy control group were excluded for identifying a current neuropsychiatric condition, which meant they were ineligible. This left 137 healthy controls (HC) for analysis. Five individuals reporting to have ADHD were excluded for having ASRS-A scores lower than the threshold (1.8% of all with ADHD), leaving 279 with ADHD. Twenty-seven were removed for stating that they took ADHD medication but not providing adherence information. A further five were excluded for over-medicating (taking more tablets than prescribed) and 38 for having low medication adherence (taking <70% of the prescribed tablets). The final sample included 110 in the medicated ADHD group (ADHD-M) and 99 in the unmedicated ADHD group (ADHD-UM). The medicated and unmedicated individuals were grouped separately due to previous research showing medication may alter risk of behavioural addiction [10, 11, 30]. Reasons for exclusion are summarised in S2 Table. Total scores were calculated for all scales (ASRS, BIS, EDS-R, BPGS, and CIUS), and subscales for the ASRS, ICBC and EDS, along with the exercise dependence risk score of the EDS-R.

Prior to data analysis to test our hypotheses, measures were checked for normality and equality of variance. To characterise the sample and identify any group differences in demographic variables, chi-square analysis was used for categorical variables (gender, ethnicity, highest qualification, and presence of learning differences) and a one-way ANOVA with post-hoc Tukey tests was used to compare the ages of participants. Following identification of group differences, an ANCOVA was used to compare the ASRS scores across the different groups to check that the ADHD groups did have higher ASRS scores as would be expected.

To test H1, examining group differences in impulsivity and compulsivity and addictive behaviours, ANCOVA was used. For addictive behaviours, data was included only from those who had engaged in the individual behaviours as noted in the methods. Given the lack of previous research on this topic in those with ADHD, a priori power calculations were based on a medium effect size (*f* = 0.25, 1-β = 0.8, α = 0.05) and revealed that 251 participants would be needed. Additionally, given that exercise dependency can be categorized a chi-square analysis was used to assess any association between category of dependency and group.

To test H2 we first calculated Pearson’s correlation coefficients for the relationships between measures of impulsivity, compulsivity and the behavioural addictions. This was followed by blocked linear regression analysis with Block 1 containing demographic variables, Block 2 including impulsivity and compulsivity scores and the final block incorporating clinical measures (Total ASRS, ADHD medication, depression and anxiety). Regression model assumptions were met [48] with no multicollinearity or singularity observed among independent variables at any stage within the backwards model, with all tolerance values were below 0.2 or 0.1, and all variance inflation factor scores approximating 1, and none above 10. The planned regression analysis incorporated 13 independent variables. As no prior work has examined this before in those with ADHD, we did not have a specific effect sizes to use in our a priori power calculations. However, we calculated that 131 people would be needed to detect a medium effect (*f*^*2*^ = 0.15, 1-β = 0.8, α = 0.05). For all tests conducted, effect size was reported (Chi-square = Cramer’s V, ANOVA/ANCOVA = *ηp*^*2*^, regression = regression coefficient B).

## Results

The final sample comprised responses from 346 participants. Categorical demographic characteristics are shown in Table 1. All groups had more females than males, however, those in the ADHD groups were significant more likely to have a higher proportion of females. Similarly, there were group differences in ethnicity with more individuals reporting a non-white ethnicity in the HC group compared to the ADHD groups. There were no significant differences in education level between groups, as evidenced by no significant association between group and highest qualification held and no significant group differences in number of years in post-compulsory education (HC 5.20±3.80, ADHD-M 4.71±3.07, ADHD-UM 5.60±7.35, *F*(2, 302) = 0.757, *p* = 0.470, ηp^2^ = 0.051). As would be expected, having a learning difference (LD) was significantly associated with having ADHD, with individuals with ADHD having a greater likelihood of having dyslexia, dyscalculia, SLD or another learning difference, compared to those without ADHD. Finally, the average age of participants was significantly different between the HC (M±SD, 40.06±14.74), ADHD-M (34.30±9.59) and ADHD-UM groups (34.36±10.51), *F*(2, 342) = 9.259, *p* <0.001, ηp^2^ = 0.005. Post-hoc Tukey tests revealed that the HC group differed significantly (*p* <0.001) from both ADHD groups, while the ADHD groups did not differ from each other.

**Table 1:** Sample characterisation of the three groups of participants: Healthy Controls (HC), those with a diagnosis of ADHD and receiving medication (ADHD-M) and those not receiving medication (ADHD-UM).

	HC	ADHD-M	ADHD-UM	χ^2^	df	*p*	*Cramer’s V*
	(N = 137)	(N = 110)	(N = 99)				
	N (%)	N (%)	N (%)				
Gender^1^							
Female	83 (60.6)	96 (87.3)	85 (85.9)	35.260	2	<0.001	0.323
Male	51 (37.2)	12 (10.9)	10 (10.1)				
Other/	3 (2.2)	2 (1.8)	4 (4)				
Prefer not to say							
Ethnicity^2^				18.608	2	<0.001	0.234
White	114 (83.2)	105 (95.5)	94 (94.9)				
BAME	21 (15.3)	5 (4.5)	1 (1)				
Other/Prefer not to say	2 (1.5)	0 (0)	4 (4)				
Highest Qualification				19.519	12	0.077	0.168
GCSE	8 (5.8)	13 (11.8)	12 (12.1)				
A levels	18 (13.1)	23 (20.9)	20 (20.2)				
Undergraduate Degree	54 (39.4)	24 (30.9)	30 (30.3)				
Higher Degree	29 (21.2)	20 (18.2)	11 (11.1)				
Professional	16 (11.7)	5 (4.5)	12 (12.1)				
Other	2 (1.5)	4 (3.6)	2 (1.0)				
None	10 (7.3)	11 (10)	13 (13.1)				
Learning Differences^3^				45.275	2	<0.001	0.364
Dyslexia	4 (2.9)	21 (19.1)	14 (14.1)				
Dyscalculia	0(0)	10 (9.1)	11 (11.1)				
SLD/Other	1(0.7)	15 (13.7)	12 (12.2)				
None	132(96.4)	73 (66.4)	69 (69.7)				

^1^Chi-square with male and female only to avoid violation of test assumption of expected cell counts.

^2^ Chi-square with white and BAME only to avoid violation of test assumption of expected cell counts.

3Given that individuals could report multiple types of learning difference (LD), chi-square was conducted comparing no reported LD with a reported difference rather than considering individual types of LD.

Table 2 shows the scores on the ASRS for the three groups. To compare groups an ANCOVA was used with gender (male, female), age, ethnicity (BAME, white) and presence of a LD as covariates. As expected, both ADHD groups scored significantly higher than the HC group on both subscales, indicating greater inattention and hyperactive/impulsive symptoms in the groups with ADHD. There were no differences between the two ADHD groups.

**Table 2:** ASRS scores for the three groups reveal that those with ADHD score higher than those without.

ASRS Subscale	HC (N = 132)	ADHD-M (N = 108)	ADHD-UM (N = 92)	*F*	df	*p*	*ηp* ^*2*^
	M(SD)	M(SD)	M(SD)				
Inattentive (IA)	13.88 (4.81)	30.75 (3.65)	30.54 (3.47)	487.94	2, 325	<0.001	0.750
Hyperactive	11.12 (5.23)	26.16 (5.24)	26.42 (5.14)	231.18	2, 324	<0.001	0.588
Impulsive (HI)							

Within the ADHD-M group medication adherence was reported as 91.0±10.3%. Of the 110 individuals in this group, 99 (90.0%) reported taking stimulants, 9 (8.2%) reported taking non-stimulant medication and 2 (1.8%) reported taking another type of medication identified as lamotrigine and modafinil. When asked about other treatments specifically for ADHD, five (4.5%) reported ADHD-specific coaching, two (1.8%) reported counselling, three (2.7%) identified CBT and one mentioned use of meditation (0.9%) or melatonin to aid sleep (0.9%).

Across both ADHD groups, 157 (75.1%) reported at least one co-morbid condition. The number of co-morbid conditions varied from 1–9, with most individuals reporting one (N = 55, 35.0%), two (N = 55, 35.0%) or three (N = 31, 19.7%) conditions alongside ADHD. The three most reported conditions were anxiety (N = 120), depression (N = 81) and autism (N = 31). Around two-thirds received some kind of treatment for their co-morbid condition(s) (65.6%). Critically, for the current study, only a small number (N = 13) reported having a compulsive disorder with the majority identifying OCD (N = 11). Most individuals receiving treatment for a comorbid condition were receiving medication (e.g., anti-depressant), but a range of therapies were also mentioned.

### Impulsivity and compulsivity are increased in ADHD

Table 3 shows the scores for impulsivity and compulsivity measures for the three groups. ANCOVA revealed significantly higher scores for all measures of impulsivity, compulsivity and associated distress in both ADHD groups compared to the HC group. There were no differences between the ADHD groups.

**Table 3:** Impulsive and compulsive measures for the three groups from the Barret Impulsivity Scale (BIS) and the Impulsive-Compulsive Behaviours Checklist which provides measures of impulsive compulsions, compulsive impulsions and overall distress.

Measure	HC (N = 132)	ADHD-M (N = 108)	ADHD-UM (N = 92)	*F*	df	*p*	*ηp* ^*2*^
	M(SD)	M(SD)	M(SD)				
Impulsivity (BIS)	30.82 (5.85)	44.61 (5.82)	44.26 (5.26)	163.25	2, 320	<0.001	0.505
Impulsive Compulsions (IC)	25.75 (5.72)	39.31 (9.14)	40.06 (9.04)	93.02	2, 308	<0.001	0.377
Compulsive Impulsions (CI)	17.59 (4.05)	25.35 (6.80)	24.83 (6.89)	42.69	2, 320	<0.001	0.216
ICBC Distress	34.75 (2.74)	42.01 (6.49)	38.78 (6.00)	48.24	2, 295	<0.001	0.246

### Elevated behavioural addictions scores in ADHD

The number of individuals engaging in specific behaviours, and therefore answering questions related to individual behavioural addictions, varied, which had implications for the statistical power. As might be expected a large proportion reported engaging in internet use (N = 305, 88%; HC = 114, 83%; ADHD-M = 97, 88%; ADHD-UM = 94, 95%). This was followed by those reporting that they undertook exercise (N = 188, 54%; HC = 89, 65%; ADHD-M = 53, 48%; ADHD-UM = 46, 46%). Only a small proportion engaged in gambling (N = 51, 15%; HC = 19, 14%; ADHD-M = 17, 15%; ADHD-UM = 15, 15%). Scores on measures of behavioural addiction (gambling: BPGS, exercise: EDS-R, internet: CIUS) were compared between the three groups (Table 4). There were no significant differences in gambling addiction. Both ADHD groups had significantly higher CIUS scores compared to the HC group (*p* = 0.001). There were no significant differences between the two ADHD groups (*p* = 0.084). The overall EDS-R score did not differ significantly between groups. However, when considering the subscales there were significant differences in withdrawal symptoms in both ADHD groups compared to the HC group. Similarly, continuance was greater in the ADHD-M group compared to the HC group. Exercise dependency risk on the EDS-R is categorised into three distinct levels: dependent, symptomatic non-dependent and asymptomatic non-dependent. The current study only identified eight participants as dependent (1 HC, 6 ADHD-M and 1 ADHD-UM). Given the low number of dependent participants, chi-square analysis was completed on the remaining two categories to examine whether there was an association between these classes of risk and group. Analyses revealed there was an association (χ^2^(2) = 7.639, *p* = 0.022, Cramer’s V = 0.206). Restricted analyses showed no association when only the ADHD groups are considered (*p* = 0.363). There were significant associations when the HC group were analysed with the ADHD-M (*p* = 0.007) but not the ADHD-UM (*p* = 0.104), with data indicating that those in the ADHD-M group were more likely to be symptomatic non-dependent than asymptomatic non-dependent.

**Table 4:** Scores on measures of behavioural addictions and the seven subscales of the EDS-R corresponding to the seven domains of addiction in the DSM-5.

Measure	HC	ADHD-M	ADHD-UM	*F*	df	*p*	*ηp* ^*2*^
	M(SD)	M(SD)	M(SD)				
BPGS	0.47 (0.96)	1.12 (1.36)	0.87 (1.36)	0.223	2, 41	0.801	0.011
CIUS	7.78 (4.91)	13.03 (4.16)	11.39 (5.26)	50.99	2, 284	<0.001	0.264
EDS-R	37.60 (14.86)	47.06 (23.93)	43.26 (18.82)	1.64	2, 172	0.197	0.019
Withdrawal	7.12 (3.60)	9.75 (4.97)	9.73 (4.59)	4.41	2, 182	0.013^1^	0.046
Continuance	5.22 (3.15)	7.84 (4.65)	6.75 (3.89)	4.37	2, 182	0.014^2^	0.046
Tolerance	6.15 (3.20)	7.26 (4.06)	6.60 (4.20)	0.38	2, 181	0.683	0.011
Lack Control	4.47 (2.84)	5.58 (4.01)	4.64 (2.62)	0.57	2, 181	0.130	0.006
Reduce Other	4.42 (2.47)	5.46 (3.14)	5.13 (2.66)	1.32	2, 181	0.271	0.014
Time Spent	5.35 (2.81)	5.70 (3.92)	5.24 (2.80)	0.20	2, 180	0.820	0.002
Quit Intention	4.52 (2.20)	5.38 (3.33)	5.25 (3.24)	0.05	2, 178	0.607	0.006

^1^ Contrasts reveal significant differences between the two ADHD groups and HC.^2^Contrasts reveal a significant difference between HC and ADHD-M only.

### Factors associated with addictive behaviour

ADHD symptoms correlated with all addictions. Correlation analyses also revealed that impulsivity was significantly and positively correlated with all addictive behaviours. Similarly, the distress caused by impulsive and compulsive behaviours significantly correlated with all behavioural addictions. In contrast, compulsivity was significantly positively correlated with exercise and internet use behaviours only and not gambling (Table 5).

**Table 5:** Correlations between measures of impulsivity and compulsivity and those of behavioural addiction.

Measure	IA	HI	BIS	CI	IC	DIS	EDS-R	BPGS
Inattention (IA)	-							
Hyperactivity/Impulsivity (HI)	862**	-.						
Impulsivity (BIS)	.799**	.761**	-					
Compulsive Impulsions (CI)	.559**	.590**	.608**	-				
Impulsive Compulsions (IC)	.704**	.733*	.621**	.665**	-			
ICBC Distress (DIS)	.603**	.615**	.591**	.721**	.772**	-		
Exercise (EDS-R)	.246**	.290**	.265**	.186*	.284**	.275**	-	
Gambling (BPGS)	.294*	.294*.	.353*	.343*	.259	.398**	.149	-
Internet Use (CIUS)	.609**	.514**	.585**	.439**	.578**	.517**	.224**	197

*<0.05, **<0.001. The ASRS IA and HI subscales are included for completeness.

Linear regression for each behavioural addiction using a blocked design revealed further detail on what factors were associated with the addiction. For gambling, only the first model was significant i.e. demographic variables (*R*^2^ = 0.297, *F*(5, 30) = 2.531, *p* = 0.05) with learning difference being the only significant positive variable in the regression (unstandardised B = 1.495, *p* = 0.039). The remaining models were non-significant (see S3 Table for full regression model data). For exercise dependency, all models were significant (Final model: *R*^2^ = 0.220, *F*(12, 137) = 2.974, *p*<0.001 –see S4 Table for full models). In all models, the presence of a learning difference was a significant positive independent variable (final model unstandardised B = 13.294, *p* = 0.003) and the final model also revealed the presence of anxiety to be a significant negative individual variable (unstandardised B = -9.801, *p* = 0.044), indicating that those with learning differences score over thirteen times higher on the EDS whilst those with anxiety score almost 10 times lower. Finally, for internet use as measured by CIUS scores all three models were significant (Final model: *R*^2^ = 0.524, *F*(13, 215) = 18.218, *p*<0.001). The findings indicate that being female, non-white, and absence of a learning difference was associated with a higher score on the CIUS, along with greater impulsivity and distress caused by impulsions and compulsions. Finally, higher ADHD symptoms were a significant positive independent variable. The full model is in S5 Table.

## Discussion

We aimed to assess the relationship between impulsivity, compulsivity, and behavioural addictions in those with ADHD and a healthy control group. Our findings demonstrated that those declaring a diagnosis of ADHD reported higher levels of impulsivity and compulsivity, and the distress caused by these, supporting H1. The increased impulsivity is unsurprising given that heightened impulsivity is a core symptom of ADHD [1]. Similarly, whilst heightened compulsivity is not commonly reported in ADHD, the condition is associated with altered fronto-striatal circuit activity which is associated with compulsivity [49] and is also found to be co-morbid with some compulsive disorders [19, 20]. The heightened distress in also aligns with previous work looking at distress in ADHD which found that distress associated with symptoms of the ADHD was increased [50]. Although this part of H1 was supported by our findings, the relationship between ADHD and scores on behavioural addiction scales was more nuanced.

For exercise, there were no overall differences in EDS-R score indicating that those with ADHD were not more likely to display exercise dependency, aligning with previous work [30]. However, that same previous study reported increased withdrawal in those with ADHD taking medication compared with healthy controls. In the current study, our findings also indicated increased withdrawal, however, this was for both ADHD groups, rather than only those taking medication. Those taking medication also reported increased continuance which was not previously found. It is unclear what underlies the differences between the subscale scores in the current study and previous work, as both cohorts were self-reporting ADHD diagnosis and had a similar medication profile with around 90% taking stimulants. Despite finding no overall differences in EDS-R, those declaring an ADHD diagnosis were more likely to be in the symptomatic non-dependent group, compared to the asymptomatic non-dependent group i.e., in a more severe, albeit not dependent group. Additionally, of the 8 individuals found to be dependent i.e., exhibiting exercise dependency, 7 (88%) identified as having ADHD and the majority were medicated (78%). These findings align with previous work [30] but also indicate that this topic needs further investigation in a larger sample, ideally sufficient to include dependent individuals in a meaningful analysis. Given that exercise dependency is estimated to be found in 3–12% of the population, this would require a very large study [51].

There were no differences in problematic gambling scores as measured by the BPGS between the groups. This contradicts previous studies which have shown higher levels of problematic gambling in those with ADHD [23–25]. A likely explanation for the lack of effects here is the small number who engaged in gambling to any degree with only 51 individuals included in this analysis, of which 32 declared a diagnosis of ADHD. The further sub-division into two groups of medicated and unmedicated likely also resulted in reduced statistical power. It is possible that differences in the how ADHD groups were characterised in the different studies underpinned the lack of effect. In the present study, participants had to declare they had an existing diagnosis from a clinician and score over the threshold on the ASRS-A [39]. Whilst in one of the previous studies, diagnosis was verified with a full diagnostic and clinical interview [23], the remaining two allocated participants into the ADHD group based only on scales scores (ASRS [25] or ASRS and Wender Utah Rating Scale [24]) in a similar approach to the current study, suggesting this is not underpinning the differences. The findings for internet usage revealed higher scores on the CIUS in those declaring a diagnosis of ADHD, indicating higher levels of problematic internet use in line with previous research [26, 27]. Therefore, in terms of our H1 we have supported the results of previous work showing some increased scores for exercise and internet addiction in those considered to have ADHD, but have not found any differences in gambling, likely due to the small number of participants gambling in the current study.

Our second hypothesis was that addiction measures would be associated with impulsivity and compulsivity, and their associated distress, as has been suggested for alcohol and substance use disorders previously. We found that impulsivity significantly correlated with all addiction scales. In contrast, compulsivity was only significantly correlated with exercise and internet use. The lack of significant correlation found for gambling and compulsivity, as well as the weaker, albeit significant, correlation between gambling and impulsivity, could be due to the sample size constraints. These results are partially aligned with, and extend, previous work. For example, previous work in the general population has found both significant [32] and non-significant [52] correlations between impulsivity and exercise addiction. This study adds to the data supporting a significant correlation and extends previous work to include individuals with higher levels of impulsivity as indicated by their ADHD diagnosis and higher ASRS scores. Similarly, previous work has also reported positive correlations between impulsivity and internet addiction in those with ADHD [34] and those without [52, 53]. Additionally, several studies have found impulsivity to correlate with gambling [23, 24, 35] in those with ADHD. In terms of compulsivity, previous research examining the relationship with exercise addiction aligns with the current results [54], as do the findings for internet addiction [55, 56]. However, we would have expected to see a significant correlation for gambling based on previous research in those without ADHD [22]. Whilst the lack of effect could be due to sample size, these findings are the first, to our knowledge, that have considered compulsivity and gambling addiction in those with ADHD.

Despite largely significant correlations with addictive behaviours, predictive value of these variables in regression models was not found. For exercise dependency, the only significant variables were the presence of a learning difference and anxiety. No prior research has investigated the role of learning differences in exercise dependence, but previous research has investigated anxiety and exercise dependence in non-ADHD populations. These studies have found no relationship between the two [57] or that anxiety can predict exercise addiction albeit as a positive, rather than negative predictor [57–59]. Therefore, the current findings indicate a different direction of effect. However, it is important to acknowledge that our measure of anxiety was binary i.e. the presence or absence of anxiety which may have impacted results. As with exercise, the presence of a learning difference was significant in the regression models for gambling. To our knowledge this is the first study to reveal this. For internet use, more factors were significant within the regression models including gender and ethnicity. The association between internet addiction and being female aligns with previous work [60, 61]. Previous research into internet addiction has shown that those with dyslexia are more at risk [62]. This directly contradicts our findings which showed that learning difference was a negative predictor. However, it is worth noting that the sample used in the two studies was distinct and the measures used varied. Additionally, the previous study focused only on dyslexia, whilst our study included a broader range of learning differences. Finally, in line with the hypothesis, impulsivity and the distress caused by impulsivity and compulsivity were significant variables in the models for internet use. As with the analyses for our first hypotheses, lack of statistical power could be relevant for gambling because the number of participants engaging in gambling was below the sample size estimated to be required for a medium effect.

### Limitations

It is important to acknowledge that whilst this study has several novel findings it also has limitations. Firstly, our sample may not be truly representative of the wider population. Recruitment and data collection were online, and therefore only accessible to more digitally literate individuals. Additionally, it is well documented that women tend to take part in more online research than men [63] which may explain the higher proportion of females in our study, despite adult prevalence of ADHD being similar in males and females [64]. Similarly, most participants in the present study were white, meaning that findings may not generalise to other ethnic groups. To be considered as an ADHD participant, individuals had to confirm that they had an ADHD diagnosis from a healthcare professional and they had to have an ASRS-A score supporting this [39]. As such we did rely on self-report of diagnostic status rather than a clinical interview which would have been more robust. We also had to exclude a high number of those without a diagnosis of ADHD i.e. forming our healthy control group, for high ASRS-A scores. This could relate to the scale choice (see below) but may also reflect self-selection bias and challenges the representativeness of this control group, creating a more selective control group than we had anticipated. An alternative approach would have been to include individuals with a high ASRS score in the ADHD group or to conduct a trait-based study rather use formal diagnosis, which would only be made if functional impairment and childhood onset were confirmed. Future studies could consider these different approaches. We also did not screen for, or exclude, individuals with substance or alcohol addictions, which may have impacted the results, although none declared these when asked about health conditions. We opted to allow all individuals with ADHD to participate in the current study irrespective of co-morbid conditions given the high proportion (>75%) of individuals with ADHD who have co-morbid conditions [65]. This has resulted in a sample in which the majority reported a co-morbidity, most commonly depression and anxiety. However, 13 individuals reported compulsive conditions including OCD and a further 11 indicated the presence of borderline personality disorder (BPD) or bipolar disorder which could increase impulsivity. Given these individuals formed only 6% and 5% of the ADHD sample respectively, this is unlikely to have impacted the results. Whilst the presence of co-morbidities indicates an ecologically valid sample [66], it does mean that those conditions could have confounded the results. Relatedly, the presence of medications for depression and anxiety could have impacted the results, with a significant proportion in receipt of SSRI medications. However, it is noteworthy that these drugs would likely decrease impulsivity and compulsivity in those taking them and therefore the effects reported could have been underestimated due to inclusion of individuals receiving this medication [65, 67].

Secondly, our choice of measures may have impacted on the findings in the current study. As indicated above, the use of the ASRS in both those with and without ADHD is supported in previous research but some studies have indicated that it should not be used as the sole measure of ADHD [68]. Additionally, whilst the scale has high specificity, it does have lower sensitivity [42]. Furthermore, although the overall internal reliability was good in the present study, when looking at the subgroups the reliability of the screener (ASRS-A) was lower. Whilst this could result from the smaller samples combined with the small number of items, it does suggest further work should consider alternative scales. Additionally, the ICBC measure used to examine compulsivity and impulsivity is mainly limited to OCD-like compulsions rather than a trait measure of these constructs and so, again, future work could consider alternative measures. Finally, the BPGS, whilst used extensively in various groups, has not been fully validated in those with ADHD. Clearly, in the absence of a previously validated scale, this was a practical choice but further characterisation of this construct in ADHD would be helpful for future research.

In addition to limitations pertaining to representativeness and assessment measures, the number engaging gambling was very small, meaning some effects would have been missed due to low statistical power. Related to sample size, in the present study we grouped all individuals taking medication together, irrespective of whether they were taking stimulant or non-stimulant medication due to the small number receiving non-stimulants. Future research should consider separating these two classes of medication, something not possible with the current sample size. This is particularly important given that medication status seemed to confer some differences in terms of scores on exercise addiction measures in this study and previous work [30]. Finally, the design of the study carried some limitations. We used a cross-sectional survey which means that no conclusions about causality can be made. It was also entirely quantitative and therefore, cannot provide us with the rich understanding of lived experiences which may be possible with qualitative approaches.

In conclusion, the current study has demonstrated that those declaring an existing ADHD diagnosis report higher levels of both impulsivity and compulsivity, as well as associated distress, compared to those without a diagnosis. We replicated previous work indicating that those with ADHD, score more highly on measures of problematic internet use and exhibit higher scores on some components of exercise addiction as well as category of risk for this tentative addiction. The latter is particularly noteworthy given that exercise is a recommended approach to managing ADHD [8]. Whilst more research is clearly needed in this area, based on this work, it would be pertinent for clinicians to discuss levels of exercise and be aware of any problematic behaviours that might emerge. Although impulsivity and compulsivity showed correlations with addictive behaviours and, despite previous research indicating that impulsivity and compulsivity may contribute to addictive behaviours, we did not find either variable to be significant in regression models of these addictions in this study. The distress caused by them was significantly associated with internet use difficulties in regression models. Further research is clearly needed to unpick the relationship between impulsivity, compulsivity, and addictive behaviours in those with ADHD.

## Supporting information

S1 Table Cronbach’s alpha values for scales for the overall sample (All) and separated into those with ADHD and without (HC). Additionally, those with ADHD are divided into those on (ADHD-M) and off medication (ADHD-UM) for completeness.
(DOCX)

S2 Table After an initial four participants were removed for not providing age information, 693 remained before exclusions were made as follows. (DOCX)

S3 Table Full regression results for problem gambling as measured by the BPGS. (DOCX)

S4 Table Full regression results for exercise dependency as measured by the EDS. (DOCX)

S5 Table Full regression results for problematic internet use as measured by the CIUS. (DOCX)

## References

[1] American Psychiatric Association. Diagnostic and statistical manual of mental disorders 5th TR ed2022.

[2] Song P, Zha M, Yang Q, Zhang Y, Li X, Rudan I. The prevalence of adult attention-deficit hyperactivity disorder: A global systematic review and meta-analysis. J Glob Health. 2021;11:04009. Epub 20210211. doi: 10.7189/jogh.11.04009 ; PubMed Central PMCID: PMC7916320. 33692893 PMC7916320

[3] Quintero J, Morales I, Vera R, Zuluaga P, Fernández A. The impact of adult ADHD in the quality of life profile. Journal of attention disorders. 2019;23(9):1007–16. doi: 10.1177/1087054717733046 28974134

[4] Hechtman L, Swanson JM, Sibley MH, Stehli A, Owens EB, Mitchell JT, et al. Functional adult outcomes 16 years after childhood diagnosis of attention-deficit/hyperactivity disorder: MTA results. Journal of the American Academy of Child & Adolescent Psychiatry. 2016;55(11):945–52. e2. 10.1016/j.jaac.2016.07.774 PMC5113724 27806862

[5] Charach A, Yeung E, Climans T, Lillie E. Childhood attention-deficit/hyperactivity disorder and future substance use disorders: comparative meta-analyses. J Am Acad Child Adolesc Psychiatry. 2011;50(1):9–21. Epub 20101203. doi: 10.1016/j.jaac.2010.09.019 . 21156266

[6] Adisetiyo V, Gray KM. Neuroimaging the neural correlates of increased risk for substance use disorders in attention-deficit/hyperactivity disorder-A systematic review. Am J Addict. 2017;26(2):99–111. Epub 20170120. doi: 10.1111/ajad.12500 . 28106934

[7] Choi WS, Woo YS, Wang SM, Lim HK, Bahk WM. The prevalence of psychiatric comorbidities in adult ADHD compared with non-ADHD populations: A systematic literature review. PLoS One. 2022;17(11):e0277175. Epub 20221104. doi: 10.1371/journal.pone.0277175 ; PubMed Central PMCID: PMC9635752. 36331985 PMC9635752

[8] NICE. Diagnosis and management of ADHD in children, young people and adults. London2019. Available from: https://www.nice.org.uk/guidance/ng87/chapter/Recommendations#managing-adhd.

[9] Humphreys KL, Eng T, Lee SS. Stimulant medication and substance use outcomes: a meta-analysis. JAMA Psychiatry. 2013;70(7):740–9. doi: 10.1001/jamapsychiatry.2013.1273 ; PubMed Central PMCID: PMC6688478. 23754458 PMC6688478

[10] Biederman J, Wilens T, Mick E, Spencer T, Faraone SV. Pharmacotherapy of attention-deficit/hyperactivity disorder reduces risk for substance use disorder. Pediatrics. 1999;104(2):e20–e. doi: 10.1542/peds.104.2.e20 10429138

[11] Turner AC, Stramek A, Kraev I, Stewart MG, Overton PG, Dommett EJ. Repeated intermittent oral amphetamine administration results in locomotor tolerance not sensitization. Journal of Psychopharmacology. 2018;32(8):949–54. doi: 10.1177/0269881118763984 29580130

[12] Dekkers TJ, Popma A, Agelink van Rentergem JA, Bexkens A, Huizenga HM. Risky decision making in Attention-Deficit/Hyperactivity Disorder: A meta-regression analysis. Clin Psychol Rev. 2016;45:1–16. Epub 20160304. doi: 10.1016/j.cpr.2016.03.001 . 26978323

[13] Daruna JH, Barnes PA. The impulsive client: Theory, research, and treatment. Washington, DC, US: American Psychological Association; 1993. p. 23–37.

[14] Verdejo-García A, Bechara A, Recknor EC, Pérez-García M. Negative emotion-driven impulsivity predicts substance dependence problems. Drug and Alcohol Dependence. 2007;91(2–3):213–9. doi: 10.1016/j.drugalcdep.2007.05.025 17629632

[15] Koob GF, Volkow ND. Neurobiology of addiction: a neurocircuitry analysis. The Lancet Psychiatry. 2016;3(8):760–73. doi: 10.1016/S2215-0366(16)00104-8 27475769 PMC6135092

[16] Luigjes J, Lorenzetti V, de Haan S, Youssef GJ, Murawski C, Sjoerds Z, et al. Defining compulsive behavior. Neuropsychology review. 2019;29:4–13. doi: 10.1007/s11065-019-09404-9 31016439 PMC6499743

[17] Dalley JW, Everitt BJ, Robbins TW. Impulsivity, compulsivity, and top-down cognitive control. Neuron. 2011;69(4):680–94. doi: 10.1016/j.neuron.2011.01.020 21338879

[18] Yang Z, Wu H, Lee PH, Tsetsos F, Davis LK, Yu D, et al. Investigating Shared Genetic Basis Across Tourette Syndrome and Comorbid Neurodevelopmental Disorders Along the Impulsivity-Compulsivity Spectrum. Biol Psychiatry. 2021;90(5):317–27. Epub 20210108. doi: 10.1016/j.biopsych.2020.12.028 ; PubMed Central PMCID: PMC9152955. 33714545 PMC9152955

[19] Miyauchi M, Matsuura N, Mukai K, Hashimoto T, Ogino S, Yamanishi K, et al. A prospective investigation of impacts of comorbid attention deficit hyperactivity disorder (ADHD) on clinical features and long-term treatment response in adult patients with obsessive-compulsive disorder (OCD). Compr Psychiatry. 2023;125:152401. Epub 20230713. doi: 10.1016/j.comppsych.2023.152401 . 37454485

[20] Grassi G, Moradei C, Cecchelli C, van Ameringen M. Who really hoards? Hoarding symptoms in adults with attention-deficit hyperactivity disorder (ADHD), obsessive-compulsive disorder (OCD) and healthy controls. J Psychiatr Res. 2023;166:74–9. Epub 20230917. doi: 10.1016/j.jpsychires.2023.09.006 . 37741062

[21] Weinstein A, Lejoyeux M. Neurobiological mechanisms underlying internet gaming disorder Dialogues Clin Neurosci. 2020;22(2):113–26. doi: 10.31887/DCNS.2020.22.2/aweinstein ; PubMed Central PMCID: PMC7366941. 32699511 PMC7366941

[22] Lee RSC, Hoppenbrouwers S, Franken I. A Systematic Meta-Review of Impulsivity and Compulsivity in Addictive Behaviors. Neuropsychol Rev. 2019;29(1):14–26. Epub 20190330. doi: 10.1007/s11065-019-09402-x . 30927147

[23] Dai Z, Harrow S-E, Song X, Rucklidge JJ, Grace RC. Gambling, delay, and probability discounting in adults with and without ADHD. Journal of attention disorders. 2016;20(11):968–78. doi: 10.1177/1087054713496461 23966350

[24] Romo L, Rémond J-J, Coeffec A, Kotbagi G, Plantey S, Boz F, et al. Gambling and Attention Deficit Hyperactivity Disorders (ADHD) in a population of french students. Journal of Gambling Studies. 2015;31:1261–72. doi: 10.1007/s10899-014-9515-9 25466366

[25] Waluk O, Youssef G, Dowling N. The relationship between problem gambling and attention deficit hyperactivity disorder. Journal of Gambling Studies. 2016;32:591–604. doi: 10.1007/s10899-015-9564-8 26271807

[26] Ho RC, Zhang MW, Tsang TY, Toh AH, Pan F, Lu Y, et al. The association between internet addiction and psychiatric co-morbidity: a meta-analysis. BMC psychiatry. 2014;14(1):1–10. doi: 10.1186/1471-244X-14-183 24947851 PMC4082374

[27] Yen J-Y, Liu T-L, Wang P-W, Chen C-S, Yen C-F, Ko C-H. Association between Internet gaming disorder and adult attention deficit and hyperactivity disorder and their correlates: Impulsivity and hostility. Addictive behaviors. 2017;64:308–13. doi: 10.1016/j.addbeh.2016.04.024 27179391

[28] Dullur P, Krishnan V, Diaz AM. A systematic review on the intersection of attention-deficit hyperactivity disorder and gaming disorder. Journal of Psychiatric Research. 2021;133:212–22. doi: 10.1016/j.jpsychires.2020.12.026 33360866

[29] Romo L, Ladner J, Kotbagi G, Morvan Y, Saleh D, Tavolacci MP, et al. Attention-deficit hyperactivity disorder and addictions (substance and behavioral): Prevalence and characteristics in a multicenter study in France. Journal of Behavioral Addictions. 2018;7(3):743–51. doi: 10.1556/2006.7.2018.58 30010409 PMC6426372

[30] Popat P, Dinu LM, Runswick O, Findon JL, Dommett EJ. Investigating the relationship between attention-deficit hyperactivity disorder, obligatory exercise and exercise addiction. International Journal of Mental Health and Addiction. 2021:1–13.

[31] Colledge F, Buchner U, Schmidt A, Wiesbeck G, Lang U, Pühse U, et al. Individuals at Risk of Exercise Addiction Have Higher Scores for Depression, ADHD, and Childhood Trauma. Frontiers in sports and active living. 2022;3:403. doi: 10.3389/fspor.2021.761844 35156014 PMC8825800

[32] Demetrovics Z, van den Brink W, Paksi B, Horváth Z, Maraz A. Relating Compulsivity and Impulsivity With Severity of Behavioral Addictions: A Dynamic Interpretation of Large-Scale Cross-Sectional Findings. Front Psychiatry. 2022;13:831992. Epub 20220617. doi: 10.3389/fpsyt.2022.831992 ; PubMed Central PMCID: PMC9248365. 35782446 PMC9248365

[33] Cabelguen C, Rocher B, Leboucher J, Schreck B, Challet-Bouju G, Hardouin J-B, et al. Attention deficit hyperactivity disorder and gaming disorder: Frequency and associated factors in a clinical sample of patients with Gaming Disorder. Journal of behavioral addictions. 2021;10(4):1061–7. doi: 10.1556/2006.2021.00074 34710057 PMC8987425

[34] Li W, Zhang W, Xiao L, Nie J. The association of Internet addiction symptoms with impulsiveness, loneliness, novelty seeking and behavioral inhibition system among adults with attention-deficit/hyperactivity disorder (ADHD). Psychiatry research. 2016;243:357–64. 10.1016/j.psychres.2016.02.020 27449004

[35] Mestre-Bach G, Steward T, Potenza MN, Granero R, Fernández-Aranda F, Mena-Moreno T, et al. The role of ADHD symptomatology and emotion dysregulation in gambling disorder. Journal of Attention Disorders. 2021;25(9):1230–9. doi: 10.1177/1087054719894378 31884864

[36] Safren SA, Duran P, Yovel I, Perlman CA, Sprich S. Medication adherence in psychopharmacologically treated adults with ADHD. Journal of attention disorders. 2007;10(3):257–60. doi: 10.1177/1087054706292165 17242421

[37] Kessler RC, Adler L, Ames M, Demler O, Faraone S, Hiripi E, et al. The World Health Organization Adult ADHD Self-Report Scale (ASRS): a short screening scale for use in the general population. Psychological medicine. 2005;35(2):245–56. doi: 10.1017/s0033291704002892 15841682

[38] Chamberlain SR, Cortese S, Grant JE. Screening for adult ADHD using brief rating tools: What can we conclude from a positive screen? Some caveats. Compr Psychiatry. 2021;106:152224. Epub 20210201. doi: 10.1016/j.comppsych.2021.152224 ; PubMed Central PMCID: PMC7116749. 33581449 PMC7116749

[39] Kessler RC, Adler LA, Gruber MJ, Sarawate CA, Spencer T, Van Brunt DL. Validity of the World Health Organization Adult ADHD Self‐Report Scale (ASRS) Screener in a representative sample of health plan members. International journal of methods in psychiatric research. 2007;16(2):52–65. doi: 10.1002/mpr.208 17623385 PMC2044504

[40] Lundin A, Kosidou K, Dalman C. Testing the Discriminant and Convergent Validity of the World Health Organization Six-Item Adult ADHD Self-Report Scale Screener Using the Stockholm Public Health Cohort. Journal of Attention Disorders. 2017;23(10):1170–7. doi: 10.1177/1087054717735381 29073818

[41] Ágoston C, Urbán R, Horváth Z, van den Brink W, Demetrovics Z. Self-Medication of ADHD Symptoms: Does Caffeine Have a Role? Front Psychiatry. 2022;13:813545. Epub 20220203. doi: 10.3389/fpsyt.2022.813545 ; PubMed Central PMCID: PMC8850715. 35185656 PMC8850715

[42] Stickley A, Leinsalu M, Ruchkin V, Oh H, Narita Z, Koyanagi A. Attention-deficit/hyperactivity disorder symptoms and perceived mental health discrimination in adults in the general population. European Psychiatry. 2019;56:91–6. doi: 10.1016/j.eurpsy.2018.12.004 30654318

[43] Guo K, Youssef GJ, Dawson A, Parkes L, Oostermeijer S, López-Solà C, et al. A psychometric validation study of the Impulsive-Compulsive Behaviours Checklist: A transdiagnostic tool for addictive and compulsive behaviours. Addict Behav. 2017;67:26–33. Epub 20161202. doi: 10.1016/j.addbeh.2016.11.021 . 27987424

[44] Spinella M. Normative data and a short form of the Barratt Impulsiveness Scale. Int J Neurosci. 2007;117(3):359–68. doi: 10.1080/00207450600588881 . 17365120

[45] Downs DS, Hausenblas HA, Nigg CR. Factorial validity and psychometric examination of the Exercise Dependence Scale-Revised. Measurement in physical education and exercise science. 2004;8(4):183–201.

[46] Volberg RA, Williams RJ. Developing a brief problem gambling screen using clinically validated samples of at-risk, problem and pathological gamblers. Health Sciences, 2011.

[47] Besser B, Rumpf H-J, Bischof A, Meerkerk G-J, Higuchi S, Bischof G. Internet-related disorders: development of the short compulsive internet use scale. Cyberpsychology, Behavior, and Social Networking. 2017;20(11):709–17. doi: 10.1089/cyber.2017.0260 29125788

[48] Field A. Discovering statistics using IBM SPSS statistics: Sage; 2013.

[49] Naaijen J, Lythgoe DJ, Amiri H, Buitelaar JK, Glennon JC. Fronto-striatal glutamatergic compounds in compulsive and impulsive syndromes: A review of magnetic resonance spectroscopy studies. Neuroscience & Biobehavioral Reviews. 2015;52:74–88. doi: 10.1016/j.neubiorev.2015.02.009 25712432

[50] Nicastro R, Desseilles M, Prada P, Weibel S, Perroud N, Gex-Fabry M. Subjective Distress Associated with Adult ADHD: evaluation of a new self-report. Atten Defic Hyperact Disord. 2018;10(1):77–86. Epub 20170610. doi: 10.1007/s12402-017-0234-9 . 28601956

[51] Orhan S, Yücel AS, Sadeq BJ, Orhan E. Investigation of the exercise dependence of athletes doing kickboxing, taekwondo, and muay thai. Sports. 2019;7(2):52. doi: 10.3390/sports7020052 30823528 PMC6409933

[52] Forsén Mantilla E, Clinton D, Monell E, Levallius J, Birgegård A. Impulsivity and compulsivity as parallel mediators of emotion dysregulation in eating‐related addictive‐like behaviors, alcohol use, and compulsive exercise. Brain and Behavior. 2022;12(1):e2458. doi: 10.1002/brb3.2458 34928542 PMC8785615

[53] Leeman RF, Potenza MN. Similarities and differences between pathological gambling and substance use disorders: a focus on impulsivity and compulsivity. Psychopharmacology. 2012;219:469–90. doi: 10.1007/s00213-011-2550-7 22057662 PMC3249521

[54] Chamberlain SR, Grant JE. Is problematic exercise really problematic? A dimensional approach. CNS Spectr. 2020;25(1):64–70. doi: 10.1017/S1092852919000762 ; PubMed Central PMCID: PMC7002162. 30915933 PMC7002162

[55] Ioannidis K, Chamberlain SR, Treder MS, Kiraly F, Leppink EW, Redden SA, et al. Problematic internet use (PIU): Associations with the impulsive-compulsive spectrum. An application of machine learning in psychiatry. J Psychiatr Res. 2016;83:94–102. Epub 20160815. doi: 10.1016/j.jpsychires.2016.08.010 ; PubMed Central PMCID: PMC5119576. 27580487 PMC5119576

[56] Tiego J, Lochner C, Ioannidis K, Brand M, Stein DJ, Yücel M, et al. Problematic use of the Internet is a unidimensional quasi-trait with impulsive and compulsive subtypes. BMC Psychiatry. 2019;19(1):348. Epub 20191108. doi: 10.1186/s12888-019-2352-8 ; PubMed Central PMCID: PMC6839143. 31703666 PMC6839143

[57] Levit M, Weinstein A, Weinstein Y, Tzur-Bitan D, Weinstein A. A study on the relationship between exercise addiction, abnormal eating attitudes, anxiety and depression among athletes in Israel. J Behav Addict. 2018;7(3):800–5. Epub 20180921. doi: 10.1556/2006.7.2018.83 ; PubMed Central PMCID: PMC6426363. 30238779 PMC6426363

[58] Lukács A, Sasvári P, Varga B, Mayer K. Exercise addiction and its related factors in amateur runners. J Behav Addict. 2019;8(2):343–9. Epub 20190531. doi: 10.1556/2006.8.2019.28 ; PubMed Central PMCID: PMC7044555. 31146551 PMC7044555

[59] Weinstein A, Maayan G, Weinstein Y. A study on the relationship between compulsive exercise, depression and anxiety. J Behav Addict. 2015;4(4):315–8. doi: 10.1556/2006.4.2015.034 ; PubMed Central PMCID: PMC4712766. 26690627 PMC4712766

[60] Choi SW, Kim DJ, Choi JS, Ahn H, Choi EJ, Song WY, et al. Comparison of risk and protective factors associated with smartphone addiction and Internet addiction. J Behav Addict. 2015;4(4):308–14. doi: 10.1556/2006.4.2015.043 ; PubMed Central PMCID: PMC4712765. 26690626 PMC4712765

[61] Haroon MZ, Zeb Z, Javed Z, Awan Z, Aftab Z, Talat W. Internet Addiction In Medical Students. J Ayub Med Coll Abbottabad. 2018;30(Suppl 1)(4):S659-s63. . 30838826

[62] Kumar S, Jackson S, Petronzi D. A preliminary study into internet related addictions among adults with dyslexia. PLoS One. 2023;18(2):e0280555. Epub 20230224. doi: 10.1371/journal.pone.0280555 ; PubMed Central PMCID: PMC9955639. 36827334 PMC9955639

[63] Waters LA, Galichet B, Owen N, Eakin E. Who participates in physical activity intervention trials? Journal of physical activity and health. 2011;8(1):85–103. doi: 10.1123/jpah.8.1.85 21297189

[64] Kooij J, Bijlenga D, Salerno L, Jaeschke R, Bitter I, Balazs J, et al. Updated European Consensus Statement on diagnosis and treatment of adult ADHD. European psychiatry. 2019;56(1):14–34. doi: 10.1016/j.eurpsy.2018.11.001 30453134

[65] Danielson ML, Claussen AH, Bitsko RH, Katz SM, Newsome K, Blumberg SJ, et al. ADHD Prevalence Among U.S. Children and Adolescents in 2022: Diagnosis, Severity, Co-Occurring Disorders, and Treatment. J Clin Child Adolesc Psychol. 2024;53(3):343–60. Epub 20240522. doi: 10.1080/15374416.2024.2335625 ; PubMed Central PMCID: PMC11334226. 38778436 PMC11334226

[66] Pliszka SR. Comorbidity of attention-deficit/hyperactivity disorder with psychiatric disorder: an overview. J Clin Psychiatry. 1998;59 Suppl 7:50–8. . 9680053

[67] Öğüt Ç, Sezer Ç. The Effects of Selective Serotonin Reuptake Inhibitors on Impulsivity in Young Adults with Major Depression in the Early Phase of Treatment. Turk Psikiyatri Derg. 2024;35(3):186–97. doi: 10.5080/u27423 ; PubMed Central PMCID: PMC11375738. 39224991 PMC11375738

[68] Vňuková M, Ptáček R, Děchtěrenko F, Raboch J, Anders M, Goetz M. Validity of the Czech Translation of the Adult Attention-Deficit/Hyperactivity Disorder (ADHD) Self-Report Scale (ASRS). Frontiers in Psychology. 2022;13. doi: 10.3389/fpsyg.2022.799344 35602673 PMC9118989

