# Supplementary material for: Investigating behavioural addictions in adults with and without attention deficit hyperactivity disorder
Source: PLoS One. 2025 Feb 5;20(2):e0317525. doi: 10.1371/journal.pone.0317525 (PMC11798432; doi:10.1371/journal.pone.0317525)
Supplement: S1 Table — Additionally, those with ADHD are divided into those on (ADHD-M) and off medication (ADHD-UM) for completeness. (DOCX) [file pone.0317525.s001.docx]

**Supporting Information S1. Cronbach’s alpha values for scales for the overall sample (All) and separated into those with ADHD and without (HC). Additionally, those with ADHD are divided into those on (ADHD-M) and off medication (ADHD-UM) for completeness.**

| **Scale** | **All** | **HC** | **ADHD** | **ADHD-M** | **ADHD-UM** |
| --- | --- | --- | --- | --- | --- |
| ASRS  Total  Screener  IA  HI | 0.97  0.91  0.95  0.93 | 0.84  0.56  0.78  0.78 | 0.82  0.53  0.70  0.79 | 0.84  0.57  0.72  0.82 | 0.80  0.50  0.69  0.77 |
| ICBC  Total  Impulsive-compulsions  Compulsive-impulsions | 0.93  0.91  0.86 | 0.89  0.83  0.85 | 0.87  0.83  0.80 | 0.87  0.84  0.81 | 0.87  0.83  0.81 |
| BIS-15 | 0.86 | 0.79 | 0.67 | 0.70 | 0.63 |
| EDS-R  Total  Tolerance  Withdrawal  Continuance  Lack of Control  Reduction in other activities  Time  Intention | 0.95  0.92  0.90  0.89  0.89  0.75  0.91  0.90 | 0.93  0.88  0.82  0.90  0.86  0.82  0.90  0.87 | 0.96  0.94  0.93  0.88  0.91  0.69  0.92  0.91 | 0.97  0.93  0.94  0.90  0.93  0.71  0.94  0.94 | 0.95  0.95  0.93  0.85  0.87  0.67  0.89  0.94 |
| BPGS-5 | 0.74 | 0.70 | 0.73 | 0.70 | 0.77 |
| CIUS | 0.89 | 0.87 | 0.83 | 0.82 | 0.83 |
