# Supplementary material for: Investigating behavioural addictions in adults with and without attention deficit hyperactivity disorder
Source: PLoS One. 2025 Feb 5;20(2):e0317525. doi: 10.1371/journal.pone.0317525 (PMC11798432; doi:10.1371/journal.pone.0317525)
Supplement: S2 Table — (DOCX) [file pone.0317525.s002.docx]

**Supporting Information S2. After an initial four participants were removed for not providing age information, 693 remained before exclusions were made as follows.**

|  | ADHD | HC |
| --- | --- | --- |
| Initial complete sample | 284 | 409 |
| Excluded for ASRS violation | 5 | 241 |
| Neuropsychiatric condition | N/A | 31 |
| Missing adherence data | 27 | N/A |
| Low adherence or over-medicating | 43 | N/A |
| Final sample for analysis | 209 | 137 |
