# Supplementary material for: Investigating behavioural addictions in adults with and without attention deficit hyperactivity disorder
Source: PLoS One. 2025 Feb 5;20(2):e0317525. doi: 10.1371/journal.pone.0317525 (PMC11798432; doi:10.1371/journal.pone.0317525)
Supplement: S3 Table — (DOCX) [file pone.0317525.s003.docx]

**Supporting Information S3: Full regression results for problem gambling as measured by the BPGS.**

| Independent Variables | Model *F* (*p*) | *R^2^* | Unstandardised B | *p* |
| --- | --- | --- | --- | --- |
| Block 1 | 2.53 (0.050) | 0.297 |  |  |
| Age (Years) |  |  | -0.005 | 0.789 |
| Education (Years) |  |  | -0.105 | 0.355 |
| Gender |  |  | -0.046 | 0.926 |
| Ethnicity |  |  | -0.197 | 0.790 |
| Learning Difference |  |  | 1.495 | 0.039 |
| Block 2 | 1.72 (0.135) | 0.373 |  |  |
| Age (Years) |  |  | -0.019 | 0.400 |
| Education (Years) |  |  | -0.097 | 0.444 |
| Gender |  |  | -0.021 | 0.970 |
| Ethnicity |  |  | -0.112 | 0.885 |
| Learning Difference |  |  | 1.833 | 0.026 |
| BIS |  |  | 0.046 | 0.170 |
| Compulsive Impulsions (CI) |  |  | -0.019 | 0.714 |
| Impulsive Compulsions (IC) |  |  | -0.058 | 0.243 |
| ICBC Distress |  |  | 0.032 | 569 |
| Block 3 | 1.41 (0.231) | 0.455 |  |  |
| Age (Years) |  |  | -0.015 | 0.556 |
| Education (Years) |  |  | -0.074 | 0.569 |
| Gender |  |  | 0.022 | 0.974 |
| Ethnicity |  |  | -0.356 | 0.661 |
| Learning Difference |  |  | 1.797 | 0.046 |
| BIS |  |  | 0.009 | 0.862 |
| Compulsive Impulsions (CI) |  |  | -0.019 | 0.748 |
| Impulsive Compulsions (IC) |  |  | -0.116 | 0.070 |
| ICBC Distress |  |  | 0.076 | 0.243 |
| ASRS |  |  | 0.049 | 0.279 |
| ADHD Medication |  |  | -0.015 | 0.980 |
| Depression |  |  | 1.258 | 0.199 |
| Anxiety |  |  | -1.117 | 0.153 |
