# Supplementary material for: Investigating behavioural addictions in adults with and without attention deficit hyperactivity disorder
Source: PLoS One. 2025 Feb 5;20(2):e0317525. doi: 10.1371/journal.pone.0317525 (PMC11798432; doi:10.1371/journal.pone.0317525)
Supplement: S4 Table — (DOCX) [file pone.0317525.s004.docx]

**Supporting Information S4. Full regression results for exercise dependency as measured by the EDS.**

| Independent Variables | Model *F* (*p*) | *R^2^* | Unstandardised B | *p* |
| --- | --- | --- | --- | --- |
| Block 1 | 4.18 (0.001) | 0.126 |  |  |
| Age (Years) |  |  | -0.100 | 0.465 |
| Education (Years) |  |  | -0.644 | 0.200 |
| Gender |  |  | 4.543 | 0.255 |
| Ethnicity |  |  | 5.373 | 0.261 |
| Learning Difference |  |  | 16.177 | <0.001 |
| Block 2 | 3.56 (<0.001) | 0.185 |  |  |
| Age (Years) |  |  | -0.029 | 0.835 |
| Education (Years) |  |  | -0.230 | 0.668 |
| Gender |  |  | 6.172 | 0.121 |
| Ethnicity |  |  | 5.064 | 0.282 |
| Learning Difference |  |  | 13.054 | 0.002 |
| BIS |  |  | 0.103 | 0.677 |
| Compulsive Impulsions (CI) |  |  | -0.254 | 0.490 |
| Impulsive Compulsions (IC) |  |  | 0.340 | 0.169 |
| ICBC Distress |  |  | 0.371 | 0.369 |
| Block 3 | 2.97 (<0.001) | 0.220 |  |  |
| Age (Years) |  |  | -0.026 | 0.848 |
| Education (Years) |  |  | -0.244 | 0.653 |
| Gender |  |  | 5.233 | 0.195 |
| Ethnicity |  |  | 5.955 | 0.206 |
| Learning Difference |  |  | 13.294 | 0.003 |
| BIS |  |  | -0.084 | 0.795 |
| Compulsive Impulsions (CI) |  |  | -01.75 | 0.643 |
| Impulsive Compulsions (IC) |  |  | 0.231 | 0.405 |
| ICBC Distress |  |  | 0.403 | 0.332 |
| ASRS |  |  | 0.250 | 0.172 |
| ADHD Medication |  |  | 1.462 | 0.729 |
| Depression |  |  | -0.352 | 0.950 |
| Anxiety |  |  | -9.801 | 0.044 |

Gender was coded such female = 0, male = 1; Learning Difference (LD) was coded as no LD = 0, LD = 1; Ethnicity coded as 0 = White, 1 = Non-white; ADHD medication was coded as 0 = No medication, 1 = Medication; Depression & Anxiety were coded as 0 = Not present, 1 = Present.
