# Supplementary material for: Investigating behavioural addictions in adults with and without attention deficit hyperactivity disorder
Source: PLoS One. 2025 Feb 5;20(2):e0317525. doi: 10.1371/journal.pone.0317525 (PMC11798432; doi:10.1371/journal.pone.0317525)
Supplement: S5 Table — (DOCX) [file pone.0317525.s005.docx]

**Supporting Information S5: Full regression results for problematic internet use as measured by the CIUS.**

| Independent Variables | Model *F* (*p*) | *R^2^* | Unstandardised B | *p* |
| --- | --- | --- | --- | --- |
| Block 1 | 7.77 (<0.001) | 0.148 |  |  |
| Age (Years) |  |  | -0.059 | 0.062 |
| Education (Years) |  |  | -0.117 | 0.223 |
| Gender |  |  | -4.021 | <0.001 |
| Ethnicity |  |  | 1.429 | 0.245 |
| Learning Difference |  |  | 0.287 | 0.752 |
| Block 2 | 25.40 (<0.001) | 0.511 |  |  |
| Age (Years) |  |  | -0.020 | 0.413 |
| Education (Years) |  |  | 0.088 | 0.249 |
| Gender |  |  | -1.898 | 0.010 |
| Ethnicity |  |  | 2.574 | 0.007 |
| Learning Difference |  |  | -1.459 | 0.041 |
| BIS |  |  | 0.264 | <0.001 |
| Compulsive Impulsions (CI) |  |  | -0.016 | 0.798 |
| Impulsive Compulsions (IC) |  |  | 0.107 | 0.011 |
| ICBC Distress |  |  | 0.138 | 0.063 |
| Block 3 | 18.22 (<0.001) | 0.524 |  |  |
| Age (Years) |  |  | -0.022 | 0.376 |
| Education (Years) |  |  | 0.075 | 0.334 |
| Gender |  |  | -1.829 | 0.014 |
| Ethnicity |  |  | 2/729 | 0.004 |
| Learning Difference |  |  | -1.566 | 0.033 |
| BIS |  |  | 0.209 | <0.001 |
| Compulsive Impulsions (CI) |  |  | -0.012 | 0.852 |
| Impulsive Compulsions (IC) |  |  | 0.066 | 0.173 |
| ICBC Distress |  |  | 0.156 | 0.037 |
| ASRS |  |  | 0.069 | 0.044 |
| ADHD Medication |  |  | -0.949 | 0.167 |
| Depression |  |  | 0.124 | 0.151 |
| Anxiety |  |  | -0.623 | -0.827 |

Gender was coded such female = 0, male = 1; Learning Difference (LD) was coded as no LD = 0, LD = 1; Ethnicity coded as 0 = White, 1 = Non-white; ADHD medication was coded as 0 = No medication, 1 = Medication; Depression & Anxiety were coded as 0 = Not present, 1 = Present.
